# Misophonia: A Systematic Review of Current and Future Trends in This Emerging Clinical Field

**DOI:** 10.3390/ijerph19116790

**Published:** 2022-06-01

**Authors:** Antonia Ferrer-Torres, Lydia Giménez-Llort

**Affiliations:** 1 L’Alfatier-Centro Médico Psicológico, 08025 Barcelona, Spain; 2 Department of Psychiatry and Forensic Medicine, School of Medicine, Universitat Autònoma de Barcelona, 08193 Barcelona, Spain; lidia.gimenez@uab.cat; 3 Institut de Neurociències, Universitat Autònoma de Barcelona, 08193 Barcelona, Spain

**Keywords:** misophonia, epidemiology, etiology, comorbidity, treatment, diagnosis

## Abstract

Misophonia is a scarcely known disorder. This systematic review (1) offers a quantitative and qualitative analysis of the literature since 2001, (2) identifies the most relevant aspects but also controversies, (3) identifies the theoretical and methodological approaches, and (4) highlights the outstanding advances until May 2022 as well as aspects that remain unknown and deserve future research efforts. Misophonia is characterized by strong physiological, emotional, and behavioral reactions to auditory, visual, and/or kinesthetic stimuli of different nature regardless of their physical characteristics. These misophonic responses include anger, general discomfort, disgust, anxiety, and avoidance and escape behaviors, and decrease the quality of life of the people with the disorder and their relatives. There is no consensus on the diagnostic criteria yet. High comorbidity between misophonia and other psychiatric and auditory disorders is reported. Importantly, the confusion with other disorders contributes to its underdiagnosis. In recent years, assessment systems with good psychometric properties have increased considerably, as have treatment proposals. Although misophonia is not yet included in international classification systems, it is an emerging field of growing scientific and clinical interest.

## 1. Introduction

Misophonia is a complex neurophysiological and behavioral disorder of multifactorial origin and is characterized by an increased physiological and emotional response produced by intolerance to specific auditory stimuli [1,2,3,4,5]. It has also been described as a form of sound intolerance, in which hyper-reactivity and selective aversion to one type of sound are present [6]. Additionally, misophonia has been considered a new mental disorder [7,8]. There is currently debate whether misophonia is an auditory or psychiatric disorder per se. The main reason for the reluctance to include misophonia among mental disorders is the danger of stigmatizing and “pathologizing” the medical picture [9]. On the other hand, it is considered that misophonia cannot be classified as an auditory disorder since no relationship has been found between it and hearing thresholds, as the disorder can occur in people with normal hearing, with hearing loss, or with some auditory pathology [9]. Furthermore, misophonia can develop in the absence of any peripheral or central auditory pathology [10]. Additionally, the specificity of the triggering stimuli suggests that the symptoms are unlikely to be caused by an alteration of the auditory system [11]. 

**Figure 1:**
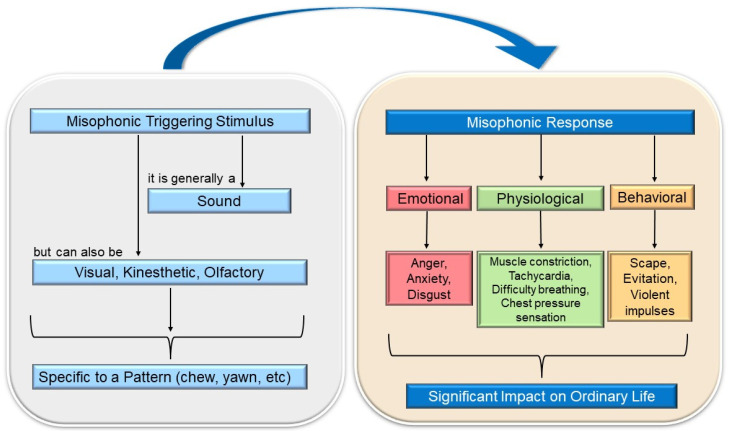
Brief explanatory diagram of misophonia (own elaboration).

Although the prevalence of misophonia is not precisely known, the figure is estimated to be close to 20% of the population [5] or 6% showing significant associated functional impairment as reported in clinical settings [12]. Misophonia may coexist with other hearing and psychiatric disorders, and it can be confused with other hearing conditions, so the percentage of affected individuals may be higher [4,12,13,14], with it being considered an underdiagnosed disorder. Misophonia has a significant impact on the sufferer’s life, as their maladaptive and avoidant behaviors interfere with the performance of work or academic tasks and cause significant impairment in their interpersonal relationships [1,6]. In response to exposure to the triggering stimulus, the individual experiences a series of physical and emotional reactions of such intensity that they affect their functionality and well-being (see Figure 1). The intensity of the misophonic responses varies depending on the emitting source, being more intense when the sound is produced by family members or acquaintances [2,9,15]. Research and health care regarding misophonia are currently scarce but slowly increasing in recent years, and the first consensus definition has been published [16]. First, there is a lack of sufficient diagnostic criteria to aid in the correct classification of misophonia and its recognition as a distinct disorder. In addition, little is known about its etiology, and insufficient assessment tools are available to measure misophonic symptoms accurately. As expected, there are no protocols for its treatment that are scientifically supported through randomized clinical trials. 

The aims of the present work were (i) to offer a quantitative and qualitative analysis of the studies on misophonia based on the data obtained in different bibliographic databases; (ii) identify the relevant known, unknown, and controversial aspects of misophonia; (iii) identify the theoretical approaches developed on misophonia; (iv) to know the methodology, types of designs used, advantages and disadvantages of each of the studies; and (v) to know the most outstanding advances that misophonia has had until 31 March 2022, as well as those aspects that remain unknown and deserve future research efforts. During the peer-review process (May 2022), some new scientific reports have been published, and because of their relevance they have been included in the discussion. 

Hence, this study presents a state of the art bibliographic review on misophonia, considering the most recent research on its etiology, epidemiology, diagnosis, comorbidity, evaluation instruments, and treatment of this disorder. 

## 2. Methods

**Figure 2:**
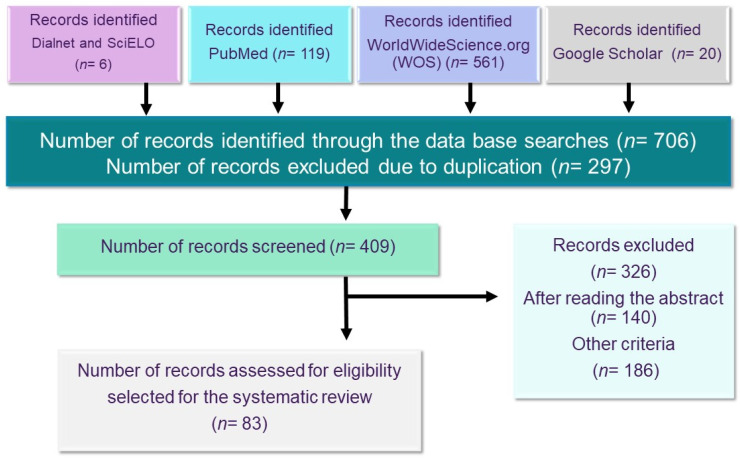
Flow chart of the systematic review on misophonia.

In the present study, a systematic review of misophonia was carried out following the guidelines of the PRISMA [17] method for its correct elaboration (see Figure 2).

The term misophonia was used to search in the following databases: Dialnet, PubMed, SciELO, and WorldWideScience.org, and also included a direct selection of 20 studies on isophonia from Google Scholar due to their scientific relevance. The PubMed database was used because it is considered the best tool for searching biomedical literature; WorldWideScience.org was also used because of its extensive database. The chronological range of the search was from 2001 to the present date, 31 March 2022. Although in other databases, including Google Scholar, misophonia has been referred to since 2001, in PubMed and WorldWideScience.org, the date of the first publication on misophonia is 2006. In SciELO, the first studies are recorded from 2015, and in Dialnet from 2017. As depicted in the flowchart, the search yielded 706 results identified as possible analysis sources. After removing 297 duplicates, the number of records to be screened was 409, but 326 were found unsuitable after reading the abstract (*n* = 140) or were not specific on the topic (*n* = 186). Therefore, the number of full-text articles assessed for eligibility selected to be included in the systematic analysis was 83.

These studies have been approached mainly by neuroscience, psychiatry, psychology, and behavioral sciences. The thematic research presented in this paper was divided into seven main areas: symptomatology, the evolution of misophonia, etiology, diagnostic criteria, comorbidity, assessment tools, and treatment options. Finally, the results section highlights those considered more interesting and points to the areas in which research on misophonia should be focused.

## 3. Results of the Bibliographic Research

### 3.1. Triggering or Misophonic Stimulus and Symptomatology

The stimuli that trigger the aversive reaction are called “triggering sounds”, “misophonic stimuli”, or “misophonic sounds” and are characterized by sharing the same pattern, regardless of the decibel level [18]. The individual’s responses following the exposure to the misophonic stimulus are referred to as “misophonic responses” [1]. These can be physical and/or emotional in nature. The former is often muscle constriction and increased heart rate [6,19]; although one may also experience a feeling of pressure in the chest, arms, head, or throughout the body, as well as increased body temperature, physical pain, or shortness of breath [2]. On the other hand, the emotional misophonic response may manifest with reactions such as anger, anxiety, disgust, avoidance behavior, escape, and/or feelings of being overwhelmed. The person suffering from misophonia recognizes that their responses are unwanted, uncontrolled, sometimes excessive, and unacceptable [20,21], but they still feel offended by those emitting the misophonic stimulus [9]. Despite this, too, they may have violent impulses toward the source of the misophonic sound [22]. In fact, anger is the primary emotional response to misophonia. One study reported that irritation (59.9%) was the most common misophonic response, and 28.6% of patients reported aggressive verbal behaviors. Even 16.7% admitted physical aggression towards objects [20].

The set of emotional reactions leads the individual to carry out maladaptive behaviors, such as asking the person emitting the misophonic stimulus to stop, arguing with them, or presenting an excessive desire to escape from the stimulus [23]. Avoidance behaviors are frequent, and although they provide the individual with momentary comfort, they worsen and maintain the symptomatology [24]. In addition, misophonia can contribute to the development of other health problems, such as behavioral disorders, emotional reactivity [1], difficulties in regulating emotions [15,25], and impaired quality of life [6,15]. It also appears to be common for people with misophonia to suffer abuse from others due to their symptoms and maladaptive behaviors. For example, it has been noted that misophonic patients routinely share on internet forums that they are accused of being “crazy” or “troublemakers” by people in their immediate environment [6]. 

It has been defined that the stimuli that trigger the aversive reaction called “triggering sounds” or “misophonic sounds”, are characterized by sharing the same pattern, regardless of their physical properties such as intensity, frequency, harshness, or decibel level [3]. Empirical studies have shown that misophonia is not limited to an aversion to loud, sharp, or harsh sounds; since even soft sounds can trigger the misophonic response (e.g., slurping sounds) [9]. In addition, misophonic sounds vary between people, so they are believed to be conditioned to individual, learning, and contextual differences. This indicates that the auditory stimulus does not produce the adverse reaction simply because of its sonic properties but is also significantly influenced by who or what elicits it [2]. Initially, it was proposed that the patient’s discomfort was elicited by the presence or anticipation of a specific sound produced by a person [20]. However, years later, it was found that the triggering sounds did not always come from human activities but were everyday sounds [6] influenced by the context and individual characteristics of the patient [26,27] as well as sounds emitted by animals or objects [2,5,28,29].

The most frequent triggering stimuli for misophonic symptoms are sounds emitted while eating [30] (e.g., chewing, crunching food, slurping, etc.), nasal sounds (e.g., breathing, sniffing, sneezing), and sounds made with the throat (such as throat clearing) [5,31]. Machine-related sounds are also common, such as those emitted by the computer keyboard, the ticking of the clock, the coffee maker, the stapler, or hair dryers, among others [21]. These are some examples, but it should be noted that triggering sounds are very varied and, although they are usually everyday [24,32], they are influenced by the context and the individual characteristics of the patient [27]. Recent research has found that the triggering stimulus can have different sensory modalities, not limited to sound alone [5,28,33]. That is, visual or kinesthetic stimuli related to the triggering auditory stimulus can also elicit the aversive response. For example, a person whose misophonic sound is the sound of chewing may generalize the aversive behavior to visual stimuli associated with the image of a particular food, e.g., fried food. In addition, another study has found that imagining a misophonic sound can trigger symptoms very similar to those experienced when hearing the actual sound [19]. 

Consequently, probably the best term to use is “triggering stimulus” or “misophonic stimulus”, since the concept of misophonic sound does not refer only to an auditory sensory modality. An unresolved unknown is whether the misophonic stimulus also produces aversive reactions when produced by the same patient. Although in a study with a sample of 92 misophonic patients, it was indicated that most do not experience symptoms when they themselves emit the triggering sound (e.g., chewing) [34]. 

### 3.2. The Evolution of the Study of Misophonia

**Figure 3:**
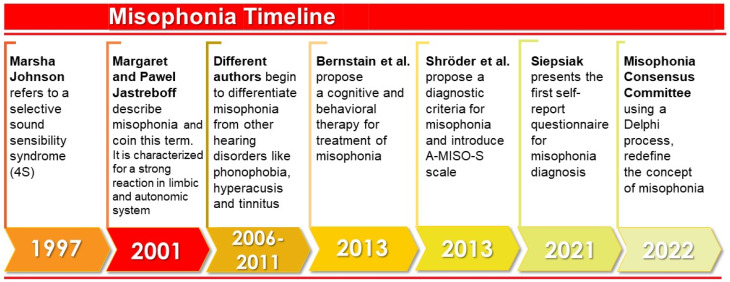
Timeline of outstanding studies related to misophonia (Own elaboration).

Figure 3 illustrates the timeline of the outstanding studies related to misophonia. In the 1990s, Marsha Johnson, an American audiologist, described for the first time what she called ‘Sensitivity to Soft Sound or Selective Sound Sensitivity Syndrome’, abbreviated as 4S syndrome [22]. With this label, she described a syndrome characterized by an evident intolerance to specific sounds unique to each individual [35]. Johnson called the annoying sound a “trigger sound”, and emphasized that associated visual or olfactory stimuli could also provoke the misophonic response [22]. 

In 2001, the authors Margaret and Pawel Jastreboff first used the term misophonia to describe a series of negative limbic and autonomic system reactions resulting from perceiving specific sounds [3]. It was suggested that this response was caused by increased functional connections between the auditory system and the limbic system. According to these authors, the auditory system functions normally, but at the behavioral level negative reactions are evoked. In other words, misophonia does not imply an increased activation of the auditory system, but the problem lies in the emotional response of the patients to the triggering sound [3].

In later years, findings made it possible to differentiate misophonia from other diagnoses such as hyperacusis or phonophobia. It was determined that misophonia was a separate disorder since, unlike hyperacusis (decreased tolerance to sound), the symptomatology was associated exclusively with one type of sound; and it was distinct from phonophobia (fear of sounds) because the primary emotional response differed [10]. Although misophonia can occur in conjunction with these pathologies, it is also seen in isolation [10].

With sufficient evidence to consider misophonia as an independent disorder, outreach efforts began in the medical community to publicize this condition and raise awareness of its impact on the individual’s life [21]. In addition, work was done to understand the prevalence of misophonia [4,5,31] and to understand the experience of misophonic individuals through case studies [2,35,36,37]. 

### 3.3. Epidemiology of Misophonia

Because the diagnostic criteria for misophonia are not established, epidemiological data are unknown, although rough estimates are available. From data collected by a tinnitus and hyperacusis center in the United States, it is estimated that 3% of the population suffers from misophonia. In addition, it was found that 92% of patients with low tolerance to sounds have misophonia, so they warn that this disorder is underdiagnosed because it can be confused with auditory disturbances [4]. 

Another figure reported in 2014 was much higher, as the incidence amounted to 19.90% of misophonia cases in a sample of nearly 500 college students in Florida [5]. Subsequently, in 2017, replicating this methodology, but accounting only for cases of misophonia with moderate severity, it was reported that 6% of their sample (*n* = 415) showed misophonic symptoms with associated functional impairment [31]. This indicates that about a quarter of the population may have misophonic symptoms, but only 6% of these individuals would meet the clinical criteria for diagnosis. 

Another later study, 2021, using the A-MISO diagnostic tool with a sample of 336 medical students from a UK university, found that clinically significant misophonic symptoms appeared to be common with 49.1% of the sample population affected [14]. A new study, also from 2021, in Ankara, Turkey, showed that misophonia was common in the general population and could cause significant disruption to daily life if not adequately treated. The results showed a prevalence of misophonia of 12.8% (*n* = 69 of 541), although 427 (78.9%) participants reported at least one sound that was distressing [38]. These results, therefore, indicated that misophonia was a phenomenon that could be experienced by a significant proportion of the population, although only a small subset experienced it severely.

The estimates differ because they are speculative rates, as the degree of misophonic symptoms has been assessed by self-administered scales, which lack a full psychiatric assessment [39]. The above figures are not generalizable but suggest that this is a real health problem that requires attention, as misophonia scores are associated with scales of functional impairment [5,15], specifically in the school or work, social, and family context [5]. 

So far, no difference in the prevalence of misophonia between sexes has been proven. Some studies have reported that a higher percentage of misophonic individuals were women [23,40], although other studies have not indicated differences [31]. It is too early to affirm or rule out that misophonia affects women differently, so this line of research should be continued by previously ruling out possible measurement biases.

### 3.4. Etiology of Misophonia

The exact causes of misophonia and the characteristics related to its evolution are not currently known. An acute and sudden onset has been observed, often in childhood or early adolescence, when the person begins to notice the sounds emitted by someone nearby and becomes hypersensitive to them. According to one paper, the mean age of onset of misophonic symptoms in a sample of 42 patients was 13 years [20]. However, a case has been reported in which the onset of misophonia was identified in a 2-year-old individual [23]. On the other hand, regarding the age of diagnosis, some authors considered that despite being a disorder that can begin in childhood, the mean age at which the diagnosis of misophonia is received is 37 years [20]. The data of another study coincided, as the mean age of 92 misophonic participants was 39 years, at which time they received health care due to the disorder [34]. It is a chronic disorder that, on average, affects patients for 30 years of their lives, as they are not usually treated [23]. In this study, the natural evolution of the disorder is a worsening of symptoms in 58% of cases, although sometimes they remain stable (25%), and rarely remit spontaneously (16.7%) [23]. In another study, 45% have worsened over time [2].

Generally, the onset of misophonia appears from an early triggering event that provokes in the individual unpleasant feelings such as disgust [9]. The onset of the disorder may be associated with unpleasant childhood memories of sounds emitted by family members (e.g., during meals) [20]. Different explanatory hypotheses for misophonia have been proposed, including traumatic events or predisposition due to other disorders. However, several studies rule out trauma as an explanation for the onset of misophonia [20,40]. 

#### 3.4.1. Genetic Predisposition Hypothesis

The genetic predisposition hypothesis suggests that obsessive-compulsive personality disorder predisposes the individual to develop misophonia [20]. Because of this relationship between obsessive-compulsive disorder and misophonia [41], and because it has been said that this personality disorder could be genetically predisposed, it was hypothesized that misophonia could also be genetically predisposed. However, although there appears to be almost 50% comorbidity between the two disorders [20], current knowledge is insufficient to determine a causal relationship between misophonia and another disorder or disease.

Within this theory, one study indicated a higher prevalence of sound intolerance among individuals with disorders such as Williams syndrome or autism spectrum disorders (ASD) [42]. Specifically, it was observed that 90% of individuals in a 118-member group with Williams syndrome showed greater sensitivity to certain sounds; and that 27% of participants with ASD also showed some degree of sensitivity to sounds greater than the control group [43].

Another study also proposed the hereditary component of misophonia, as it found that 15 members of the same family met the criteria for the diagnosis of this disorder [23]. Likewise, another study also concluded that one-third of the participants with misophonia (*n* = 301) reported a family history with the same symptomatology [30]. The fact that participants manifest misophonic symptoms during childhood or early adolescence could indicate that they learn to reproduce the behavior of other family members. Although, according to information from the participants, some of those affected had little contact with the rest of the family and still developed the disorder [23].

One study suggested that personal characteristics such as age (childhood and early adolescence), sex, and family traits during the individual’s development (e.g., a family member having misophonia) mediated the onset of misophonia [40]. However, the influence of these factors is still unclear, as in other studies, age and sex were not significantly associated with the severity of misophonia [5,34]. So far, no genetic studies have been carried out to show that auditory stimulus intolerance has a hereditary component, so this explanation does not yet have sufficient empirical support. 

#### 3.4.2. Neurobiological Alterations Hypothesis

The emotional response experienced by misophonic patients in the presence of sound raised the question of whether auditory information was processed differently or increased in these individuals. However, the symptomatology of misophonia associated with only one type of sound suggested that, if there was an anatomical alteration, it would be found in cortical areas responsible for information processing [10]. In this sense, it was observed that most people with misophonia had normal auditory sensitivity [44]. If the misophonic response was not due to an alteration of the auditory system as initially thought, it was proposed that the response of misophonic patients might be due to an alteration of the limbic system and the autonomic nervous system, which react abnormally to auditory input of normal intensity [10]. Symptoms of increased blood pressure evidence activation of the autonomic nervous system and heart rate, increased body temperature, sweating palms, physical pain, and shortness of breath after exposure to trigger stimuli [2]. The increased physiological response has been explained as a result of increased arousal of the sympathetic nervous system, which in turn produces distress in the individual [1]. Edelstein et al. [2] pioneered the study of the physiological response of people with misophonia, confirming that their reaction to the triggering sound included an increase in skin conductance, which did not occur in the presence of nonaversive stimuli of another modality [2]. This physiological reaction showed a positive and significant correlation with the intensity of the aversion indicated by the individual, whether misophonic or control. Such a finding suggested that people with misophonia manifested the same reaction as the general population had to aversive stimuli, with the difference that they experienced the discomfort much more intensely [2].

Subsequently, a study by Schröder et al. [45] used functional magnetic resonance imaging (fMRI) for the first time to localize functional neuroanatomical correlates of misophonia [45]. Compared to control patients, participants with misophonia showed increased neural activity in the visual cortex, temporal cortex, amygdala, and anterior insula. However, these results were taken as preliminary, as it was not evident whether the increased activation of these regions in patients occurred exclusively in the presence of the triggering stimuli [45]. Continuing this line of research, in 2019, the reactions of 21 patients with misophonia and 23 without were analyzed [46]. They presented signals of multimodal nature (auditory and visual) in three conditions: related to the misophonic stimulus, aversive, and neutral stimuli. Measurements of the behavioral and emotional responses of the participants were also performed to have a better understanding of the misophonic reaction. The results indicated that misophonic audiovisual stimuli caused anger, disgust, and sadness among patients with misophonia, accompanied by physiological arousal and increased activity of the right insula, right anterior cingulate cortex, and right superior temporal cortex [46]. This set of reactions occurred only during exposure to aversive stimulus cues, as the responses of patients with misophonia to neutral stimuli were the same as the rest [46]. Answering the previous unknown, these findings demonstrated that there is no permanent functional impairment but rather an exaggerated response to a specific stimulus.

From this study, the authors suggested that the insula and the right anterior cingulate cortex activity indicated the overactivation of the network responsible for the detection and selection of emotionally relevant information [46]. Since the misophonic signals were identified as salient information, there was an increase in cardiovascular arousal and other autonomic responses. On the other hand, the hyper-reactivity observed in the right superior temporal cortex would explain the attentional increase of misophonic individuals to the triggering stimulus due to the relevance of this area for auditory selective attention [46].

Overactivation of the insula had also been reported by Kumar et al. [47], who also detected abnormal connectivity between it and cortical regions such as the medial frontal, medial parietal, and medial temporal cortex [47]. The authors attributed these connectivity alterations to an abnormality in myelination in the medial frontal cortex, which results in the neuronal response responsible for misophonic patients’ physiological arousal and emotional response [47,48]. Thus, the most recent studies by Eijsker et al. [48,49] are also rather important as they investigated the neuropathology basis of the disorder and provided new findings on structural and functional brain abnormalities.

An interesting paper from 2021 studied misophonia from a motor perspective [50], where the mirror neuron system related to orofacial movements and the visual and auditory cortex could be the basis of this disorder. According to Kumar et al. [50], in people with misophonia, there was increased functional connectivity between the auditory cortex and the orofacial motor areas in response to all types of sound; in addition, there was also increased activation of the orofacial motor area in response to trigger sounds and, finally, the activation of the orofacial motor area increased in proportion to the misophonic discomfort. 

However, despite the optimism in determining these theories as to the neurobiological basis of misophonia, some authors mentioned that the lack of consensus about the diagnosis was its major limitation [51]. They also emphasized that the collaboration of psychiatrists and psychologists was indispensable in the process of participant selection in order to make a comprehensive evaluation that complements the results of the subjective scales [51]. On the other hand, other authors emphasized that anger was not a primary emotional response in misophonia, as another study with more than 300 patients reported annoyance as the primary reaction to this disorder [40]. Although it is impossible to determine that the described alterations are the biological bases causing misophonia, they are undoubtedly associated with the disorder.

#### 3.4.3. Conditioning Hypothesis

It is believed that learning may partly explain the emotional and behavioral symptoms of misophonia. In this sense, the misophonic response would be a conditioned behavior through classical conditioning: there is a neutral stimulus (e.g., chewing noise) that, after being associated with an aversive unconditioned stimulus, triggers the same unconditioned response (anger, irritation, etc.) [3]. The emotional and physiological response results from the activation of the limbic and autonomic nervous system, producing irrational reactions to the conditioned stimulus (the misophonic sound) [24]. 

At the behavioral level, patients avoided situations involving the inclusion of the misophonic sound, and in the long term, the avoidance reinforced the pathological response (since it functioned as a negative reinforcer), exacerbating the symptomatology [24]. Ultimately, this is a conditioning process in which the individual learns to respond disproportionately to an auditory stimulus. For example, one study presented 10 cases that supported the development of misophonia as classical conditioning [35]. On the other hand, it is also possible that individuals with misophonia are sensitized to the triggering sound [52]. Although the neural mechanism for sensitization in misophonia is unknown, it may be related to the long-term potentiation of limbic system neurons [2]. Similarly, Jastreboff and Jastreboff [4] considered that the triggering sound was part of a conditioned stimulus that was influenced by the context and by psychological factors [4]. In fact, these authors proposed that the treatment of misophonia should aim at desensitizing patients to the triggering stimulus; and this would be achieved after systematic exposure to the conditioned stimulus [51] despite the disagreement of Dozier, who argued that exposure could lead to a worsening of symptoms [53]. 

### 3.5. Diagnosis of Misophonia 

Misophonia is not included in any diagnostic classification, so there is no consensus on the criteria for diagnosis. However, different authors have proposed a series of diagnostic criteria based on the current body of knowledge [20,29]. 

#### 3.5.1. Diagnostic Criteria

The need for standardized diagnostic criteria stems from their importance in promoting the recognition of this disorder, the development of empirical work, and the improvement of the evaluation and treatment of misophonia [20,29,54]. Some authors suggested that misophonia should be considered an obsessive-compulsive spectrum disorder [20]. They proposed six diagnostic criteria, among which it was stated that the presence or anticipation of the sound causes an aversive physical reaction, that anger is the salient feeling, and that the sensation experienced by the patient is explained by obsessive-compulsive disorder or post-traumatic disorder [20]. On the other hand, another diagnostic proposal for misophonia was developed where it was indicated that the diagnostic criteria needed to be updated based on new scientific evidence findings [29]. For example, it was found that misophonic sound could come from any source, not strictly limited to human activities [2,5,28,29]. Thus, a triggering stimulus can be a sound emitted by animals, electronic devices, or other objects. Based on these and other considerations, an adapted and updated version of the diagnostic criteria for misophonia was proposed [29]. For example, the stimulus is considered to elicit an immediate physical reaction that is not always easy to identify [29].

#### 3.5.2. Differential Diagnosis

It should be noted that the symptomatology of misophonia may share characteristics with other psychiatric and auditory disorders or be confused with them, contributing to its underdiagnosis [4].

Table 1 shows the disorders that may be most frequently involved in the differential diagnosis of misophonia and their similarities and differences. 

### 3.6. Comorbidity

High rates of comorbidity of misophonia with mental disorders have been reported, and therefore, it has been suggested to classify misophonia as such [5,20,25,34]. In contrast, there are authors who indicated very low rates of comorbidity, arguing that misophonia is detached from psychopathological disorders and should be considered as a distinct entity [56]. The most frequent mental disorders comorbid with misophonia are depression [6], obsessive-compulsive disorder (OCD) [57,58], and anxiety [22]. Comorbidity has also been observed with neurological disorders such as Tourette’s syndrome and auditory system disorders [42,59].

#### 3.6.1. Misophonia and Associated Mental Disorders

Preliminary research states that misophonia can coexist with a wide range of psychiatric disorders [8,25]. Anxiety disorders have been shown to be often associated with misophonic symptomatology [5,31,34,60]. For example, one paper indicated significant correlations between misophonia scales with other OCD, anxiety, and depression scales [5]. This one also showed that anxiety was a significant mediator between misophonic symptomatology and anger [5]. On the other hand, another study reported that 12% of people with misophonia had a first diagnosis of anxiety disorder and that the severity of the anxiety disorder was significantly associated with the severity of misophonic symptoms [34]. Based on this relationship, the authors suggested that anxious psychopathology might contribute to the development of misophonia by predisposing the individual to be more sensitive and aware of their environment. If this hypothesis is proven, misophonia would be a consequence of the anxiety disorder due to hypervigilance of the nervous system [34]. Although this hypothesis seems logical, it should be considered that other studies indicated that more than 80% of misophonic patients do not suffer from an anxiety disorder. 

Apart from this theory, it has been suggested that the association between misophonia with anxious, obsessive, and depressive symptoms indicated phenomenological similarities that did not necessarily imply the presence of a common disorder but rather reaffirmed that misophonic symptoms were not fully explained by any of these psychopathologies, contributing to their identification as a distinct diagnostic entity [5]. In this regard, one paper describes two cases of misophonic patients treated with anxiolytic and antidepressant medications, which, despite alleviating anxious and depressive symptoms, failed to diminish their aversive reaction to triggering stimuli [2].

OCD is one of the mental disorders most frequently associated with misophonia; approximately half of the people with misophonia also have obsessive-compulsive symptomatology [15]. Based on this estimate, the authors believed that misophonia should be classified as a type of OCD [15]. Misophonia shares clinical features with OCD in that the preoccupation with the stimulus is experienced as intrusive and unwanted thoughts, and furthermore, the individual in both cases seeks strategies to avoid distress [5,31,58]. It has also been proposed that misophonic symptoms and sensory overactivity are shared in both OCD and misophonia [58]. 

One study focused on the task of analyzing the relationship between OCD symptoms and misophonia among a large sample of college students and the general population (*n* = 828) [57]. It was found that elevated OCD or misophonia symptomatology scores increased the risk of developing the other pathology. Furthermore, misophonia was significantly associated with obsessive symptoms of OCD, suggesting that obsessive thoughts were a particularly shared feature between the two disorders rather than compulsions [57].

The clinical similarity between misophonia and OCD leads some to consider that misophonic reactions are best described as a symptom of OCD, generalized anxiety disorder, or a personality disorder [61]. However, a 2018 study of nearly 400 misophonic patients showed that only 50% of the participants had comorbidity with another psychiatric disorder, whereby their condition could not be explained as an effect of another diagnostic entity [40]. Although the evidence suggests the existence of misophonia as a distinct pathology, it is important to keep in mind that comorbidity with OCD increases the severity of the condition because the symptomatology overlaps: misophonic stimuli produce a negative emotional reaction, which is reinforced by avoidance behavior, and these in turn increase the patient’s anxiety and distress [58]. This was verified in another study, which reported that misophonic patients with other comorbid conditions such as OCD or PTSD report more severe symptoms compared to those suffering exclusively from misophonia [40].

The comorbidity of post-traumatic stress disorder (PTSD) with misophonia had previously been reported in the case of one patient [2]; although he met diagnostic criteria for PTSD, the pathology did not explain the patient’s aversive response to one type of sounds. Another study with a large sample [40] noted that the rate of this comorbidity amounts to 12%, the same percentage reported for comorbidity with tinnitus. The symptomatology shared between PTSD and misophonia is the decreased reactivity threshold since, in both cases, patients experience muscle tension in response to a triggering stimulus; therefore, it was suggested that associative learning may influence the development of the conditioned response in the two disorders [40].

Moreover, several papers observed links between misophonia and other psychiatric disorders [8]. In a sample of 92 misophonic participants from Singapore, this diagnosis was found to be associated with others such as depression (52% of cases) and schizophrenia (22%) [34]. Depression was not significantly associated with the severity of misophonic symptoms, and the authors suggested that this disorder may not be relevant in the development or understanding of misophonia [34]. Instead, these results differ from other studies that reported positive and significant correlations between symptoms of depression and misophonia [5,31]. Therefore, the association of depression with this disorder should not be ruled out, but needs to be studied with greater specificity. These authors suggested that low tolerance to a sound may increase depressive symptoms, although the relationship could also be inverse, i.e., that depression predisposes the person to have greater sensitivity to certain sounds [5]. 

It should be noted that misophonia was also found to occur with other pathologies such as attention deficit disorder with and without hyperactivity (ADD/ADHD) in 12% of cases, eating disorders (8%), and selective mutism (6%) [40]. However, for the authors, none of these added disorders was sufficient to explain the misophonic symptoms [40]. Recent studies evaluated the relationship between inattention and misophonic symptoms [59]. For example, a study assessing selective attention in patients with misophonia showed that participants had a lower percentage of correct responses on the chewing sound dichotic sentence identification test [62]: suggesting that individuals with misophonia may have impaired selective attention when exposed to sounds that trigger this condition [62,63]. As can be seen, different psychopathologies have been associated with misophonia as they share clinical features and comorbidity is present in about half of the cases, so it is important that the study of this line continues [20,40].

A recent study, 2022, found two potential subgroups of individuals with misophonia: one with “pure form” misophonia with more severe misophonic symptoms but few concurrent conditions, the second subgroup showed greater numbers of concurrent conditions that may represent misophonia as a secondary phenomenon of other neuropsychiatric conditions [64]. These subgroups create a new demand: to explore the relationship between misophonia sensory symptoms, misophonia emotional reactivity/behavioral symptoms, and other related neuropsychiatric conditions, such as ASD and anxiety, with emphasis on neural mechanisms. Future work should evaluate auditory stimuli and their responses, as well as the assessment of cognitive control difficulties exhibited by individuals with misophonia to address the possible relationship between misophonia, sensory processing disorders, executive function, and state/trait anxiety levels [65].

#### 3.6.2. Misophonia and Neurological and Auditory Disorders 

Proper functioning of the central and peripheral nervous system is essential for optimal information processing. Although it appears that this system functions adequately in misophonic patients, there are cases in which a neurological disorder coexists and overlaps the symptoms [2,44,46]. Tourette’s syndrome (TS) is a neurological disorder that presents in childhood or adolescence and is characterized by the presence of motor and phonological tics. It is also considered a psychiatric disorder, because its symptoms resemble the compulsive behaviors of OCD [6]. One paper described the case of a 52-year-old man with TS who, one year before the onset of the syndrome’s symptomatology, began to develop an aversion to the sound his father made while chewing [59]. The misophonic responses were in addition to the motor tics, and this contributed to a series of behavioral problems that affected his quality of life. The authors suggested the possibility of an associated physiological disturbance between the two disorders, although this has not been demonstrated at present [59]. 

Sensory processing disorder (SPD), also called modulation disorder or sensory integration disorder, is a condition in which sensory information is inappropriately processed and organized. As a result, developmental delays, and emotional and behavioral problems such as those characteristics of autism spectrum disorders occur [66]. TPS, more common in children, is evidenced by an exaggerated or inappropriate response to the presence of various sensory stimuli [66]. For example, aversive responses to certain colors, sounds, or tactile stimuli are observed. One study showed that individuals with misophonic symptomatology also had an intolerance to tactile stimuli, suggesting a relationship between TPS and misophonia [67]. In addition, individuals with sensory intolerance had higher rates of general psychopathology, obsessive-compulsive symptoms, and poorer social and occupational functioning [67]. No cases of comorbidity between misophonia and olfactory processing disorders -such as hyperosmia- have been described, although it is likely that they are related since their neurobiological substrate is thought to be associated with alterations of the autonomic nervous system and limbic system, as occurs in misophonia [68,69,70].

When the failure of sensory processing occurs in the auditory system, it is referred to as auditory processing disorder (APD), which produces a high intolerance to sounds [67]. In the scientific literature, a case has been reported of a misophonic patient who had a previous diagnosis of APD, which alone did not explain the clinical reactions [2]. Thus, misophonia can coexist with TPA, where there is a hyper-reactivity towards loud and unexpected sounds (caused by TPA), and in addition an aversive response towards sounds of the same pattern irrespective of their physical properties (due to misophonia). The neurobiological mechanisms of TPA could be related to those of misophonia, although in the latter case, it is an overactivation of the limbic system, whereas TPA is due to a lack of maturation of the central auditory system.

In relation to the alterations of the auditory system, tinnitus and hyperacusis have a close relationship with misophonia, due to the similarity of symptomatology. It is essential to make a correct differential diagnosis, but sometimes patients present comorbidity with these hearing disorders. The association between these disorders has been confirmed in other studies, and it is estimated that 12% of patients with misophonia also have tinnitus [2,40]. The causes of these conditions differ, so they should be treated as distinct entities requiring specific treatment [27,56].

### 3.7. Instruments and Procedure for the Assessment of Misophonia

The main difficulties faced in research and health care for patients with misophonia are related to the assessment process. At present, there are very few objective tests that assess this diagnosis.

#### 3.7.1. Clinical Anamnesis

Clinical anamnesis is an essential tool for recognizing misophonic symptomatology, which will also provide information on the onset and course of the disorder, the triggering stimuli, and the patient’s emotional and physiological reactions. Authors have emphasized the importance of a complete evaluation that involves the interview of the psychiatrist and/or psychologist, to know the presence of comorbid conditions, as well as the use of drugs or psychoactive substances [24,39].

The anamnesis is also a tool commonly used in research, especially in case studies [2,35,36,37,71]. Although the structure and content of the anamnesis is variable, and depends on the needs of the practitioner, there are a number of data that should be collected. In addition to patient identification data, it is essential to collect as much detail as possible about the history and characteristics of the symptoms, in order to determine whether there are sufficient reasons to think of a pathology, and to analyze which best explains the clinical picture.

When misophonia is suspected, it is crucial to find out if there are cases of family members who have an aversion to a particular type of sound, because as previously mentioned, it is common for several members of the same family to present misophonic symptoms [20,23,40]. Other essential information is to know since when the aversive reactions to the triggering stimuli began, and how the symptomatology has evolved. The precise misophonic response of the patient must also be specified, as this varies in each individual, e.g., the specific emotions and physical sensations experienced must be recognized. In relation to the misophonic stimulus, it should be investigated whether it is exclusively auditory stimuli, or whether the clinical response has extended to other sensory modalities such as visual, kinesthetic, or olfactory [2,27,67]. Moreover, to understand the causes of the maintenance of the disorder, it is necessary to describe the patient’s behaviors and coping strategies, which will also be relevant for treatment planning [29,35].

#### 3.7.2. Audiological Evaluation

A 2021 study, which assessed (*n* = 253) misophonia with an online psychoacoustic test, suggested that such a test can be used to assess misophonia reliably and quickly [72]. However, although audiological measurement is historically a suggested component of the assessment of misophonia, there is no agreement on the protocol that should be performed [24]. The lack of consensus is due to the fact that there are no clear audiological criteria for the diagnosis of misophonia, as misophonic patients have shown both reduced levels of hearing and normal levels [4,73]. Audiological assessment includes measurement of pitch thresholds and sound discomfort levels, but since no such criteria are known to characterize misophonia, for now, assessment should focus on the clinical history and questionnaires described below.

However, even if the audiological evaluation does not in itself confirm misophonia, it may be important in making the differential diagnosis of other hearing conditions. 

If sound intolerance is not specific to the same sound pattern, it is necessary to rule out hyperacusis, and hyperacusis can be detected through audiological studies since patients with hyperacusis generally present discomfort at lower than average volume [6]. In addition, let us remember that hyperacusis and misophonia often coexist, so audiological evaluation will be substantial in these cases [2,4,40]. There are questionnaires that, together with audiological tests, allow us to know the hypersensitivity to sound, such as the hyperacusis test (THS) [74]. It is a 15-item questionnaire that evaluates three dimensions: cognitive behavior in relation to hyperacusis, somatic behavior, and emotional reactions. It is answered on a four-choice Likert-type scale (0–3), and the overall score indicates the degree of impairment. It has been adapted and translated into Spanish, showing a high internal consistency (a = 0.90) [75].

#### 3.7.3. Self-Administered Scales of Misophonia

The second type of tools most commonly used for the assessment of misophonia are self-report instruments, although most of them are not validated. The most representative ones to date are shown in Table 2.

The most recent questionnaire is the Sussex Misophonia Scale for Adolescents (SMS-Adolescent), the only one that currently assesses misophonia in children/adolescents [85]. It is an adaptation of previous version for adults of the same authors [86]. The study was conducted with a sample of 142 children (10–14 years; mean 11.72 SD 1.12; 65 females, 77 males). In addition, the study was able to demonstrate that children with misophonia showed significantly higher levels of anxiety and obsessive-compulsive traits than children without misophonia. In addition, they also showed that health-related quality of life was significantly poorer. 

#### 3.7.4. Emotional Assessment

An important component of the misophonic response is emotional, and it is therefore crucial to include such measurements in a comprehensive assessment of a patient with possible misophonia. Previous evidence indicates that misophonia causes psychological distress and impaired quality of life, so it is important to assess these variables [2,6,15,21]. Furthermore, given that the misophonic response is associated with anxious and depressive symptomatology, it is important to incorporate inventories that allow us to know the patient’s emotional state [5,31,34]. 

#### 3.7.5. Physiological Measurements

It has been shown that the patient’s physiological response is part of the misophonic symptomatology, so that measurements of nervous system activity can be useful in the diagnosis of the disorder [44]. Electroencephalography (EEG) and functional magnetic resonance imaging have been used in research to record neuronal activity [45,46], as well as measurements of galvanic skin response [2].

From the above, it would be pertinent to propose the use of the electrocardiogram (ECG), to identify the reactions of the autonomic nervous system, since it allows recording the heart rate variability (HRV) of patients and identifying the presence of alterations [87]. In this respect, one of our studies [19] has shown that in highly restrictive environmental situations, such as the one that occurred during the COVID-19 lockdown, the heart rate variability of patients with misophonia underwent changes with respect to the recordings of the same triggers previously measured. More importantly, this loss of coherence also occurred with imagined or evoked sound triggers.

Although there are no specific physiological indicators of the misophonic response, it is known that there is an increase in autonomic activity [1,6] and the identification of these reactions would favor the understanding of misophonia, as well as guide treatment. Taylor [9] proposed that, for the consolidation of misophonia as a diagnostic entity, it was important to establish precise biological and physiological indicators, which could be identified by laboratory studies for the delineation of the disorder and, subsequently, for its diagnosis [9].

### 3.8. Treatment Options 

This section presents the possible treatments that have been studied and proposed in recent years to treat misophonia. According to this literature review, there are currently no randomized controlled trials examining the efficacy of treatments for misophonia [1]. These are strategies developed through clinical practice, which have not been studied under adequate experimental conditions. Moreover, Brout et al. [1] warned of the need to recognize that data are still insufficient to classify misophonia as a psychiatric disorder, which should also be explained to the patient [1]. In the absence of a specific treatment for misophonia, a multidisciplinary approach becomes especially important for the treatment of related physical and emotional conditions [88]. 

#### 3.8.1. Audiological Treatment

Misophonia was recognized at the beginning of its study as an auditory condition, similar to tinnitus or hyperacusis. Consequently, the usual therapeutic intervention consisted of the use of devices for the retraining of the triggering sound, or portable white noise generators to minimize the concentration on misophonic sounds [22]. A clear example of the therapeutic approach from audiology is tinnitus retraining therapy (TRT), which offers different treatment protocols in which the patient is systematically exposed to the triggering sounds, with the aim of achieving habituation to it [3,4,27,54,56]. Another case study included a hybrid approach involving TRT and telecare, with good results [89]. In relation to TRT, it has been reported that more than 80% of misophonic patients in a group of 184 participants improved after this treatment [56]. In addition to the lack of scientific evidence, this strategy may help in symptom control, especially in patients who have comorbidity with tinnitus, but it does not address the complexity of misophonic responses or the causes of the syndrome. 

Similarly, Johnson [69] postulated that the most effective treatment for misophonia is educational counseling in combination with auditory therapies [69]. The latter generally consist of the use of devices, the size of hearing aids, that produce sounds that help mask aversive stimuli. There is no known support from experimental studies demonstrating the effectiveness of the therapy proposed by Johnson, yet. 

#### 3.8.2. Pharmacological Treatment

Although there is no specific pharmacological treatment for misophonia or research protocols in this regard, the use of antidepressant and anxiolytic drugs has been described to attenuate the clinical symptomatology or comorbid conditions associated with misophonia [24]. In this regard, two cases are reported of misophonic individuals who were treated with anxiolytics and antidepressants to alleviate emotional responses, although this treatment did not solve the problem, as they continued to experience clinical symptoms [2]. 

However, a recent study has reported drug use directly affecting misophonia. This particular study showed the case of a 14-year-old adolescent girl with misophonia successfully treated with fluoxetine. In the fourth month of treatment, there was a 40% decrease in the Children’s Global Assessment Scale (CGA) score [87]. In addition, another study also demonstrated that the use of methylphenidate helped an adolescent patient decrease symptoms of distractibility due to misophonia [90]. Although these types of drugs were developed to treat psychopathologies other than misophonia, they are probably useful in those patients who present association with symptoms of depression or anxiety [5,31,34]. 

There is also a case study, conducted in 2022, wherein propranolol, a β-blocker, was used to successfully treat a patient experiencing misophonia and misokinesia by completely eliminating multiple auditory and visual triggering symptoms related to eating with other people [33]. In another case study from 2022, a child with autism responded favorably to a low dose of risperidone [42]. 

As a future line of research, it would be interesting to analyze, through randomized clinical trials with large samples, the efficacy of psychotropic drugs in the treatment of misophonia.

#### 3.8.3. Cognitive-Behavioral Therapy

The main goal of this approach is to develop coping skills to prevent and respond appropriately to one’s own misophonia symptoms [1,91]. It is important to note that there are no proven psychological therapy protocols for misophonic patients yet, so this treatment approach is based for now on the general principles of cognitive-behavioral therapy [92]. 

Some authors considered cognitive behavioral therapy (CBT) to be effective in the treatment of misophonia given that sympathetic nervous system hypersensitivity may be a threshold amenable to modification through cognition and behavior [16]. In addition, CBT has been observed to support emotional and behavioral control in patients with misophonia when they hear or anticipate misophonic sounds. For example, based on CBT principles, a tailored psychoeducation course was implemented for a patient with misophonia, the aim of which was to provide information about anxiety and its relationship with the hypothalamic-pituitary-adrenal axis [22]. The assumption was that the sensitive threshold could be modified through cognitive and behavioral self-management. Treatment included identification and restructuring of negative automatic thoughts, modification of maladaptive behaviors such as avoidance, and training to control autonomic nervous system reactivity. After six treatment sessions, the patient achieved that exposure to misophonic sounds did not significantly impede her social and occupational functioning. Moreover, at the end of the intervention, a reduction in symptoms and an increase in her life satisfaction were observed, results that remained stable over four months of follow-up [22]. 

A 2015 case study showed improvement of misophonic responses with two young people after application of CBT [93]. In 2017, a study was conducted with a large sample of misophonic patients (*n* = 90) who received a treatment of eight sessions of CBT, whose efficacy was assessed using the clinical global impression of improvement scale [51]. At the end of the treatment, 48% of patients had significant clinical improvement in relation to misophonic symptoms. Furthermore, it was found that the severity of misophonia and the presence of disgust positively predicted the response to CBT. That is, patients with severe misophonia and those experiencing disgust to the triggering stimulus were more likely to respond to treatment [35]. Finally, another case study, also from 2017, in addition to CBT, included dialectical behavioral therapy (DBT) with good results arguing that this therapy might be appropriate for people with misophonia who experience intense anger responses [94].

In general, different studies have presented cases subjecting misophonic patients to different cognitive-behavioral therapy plans, using, for example, principles of exposure and response prevention [71,92,95,96]. Moreover, separate analyses have been performed for patients with tinnitus, hyperacusis, and misophonia [96,97]. These studies have been successful in increasing the level of tolerance to triggering stimuli, significantly reducing psychological distress and self-reported physiology, and significantly reducing the feeling of anger at the triggering stimulus [71,96,97]. 

#### 3.8.4. Third-Generation Therapies

Other approaches to misophonia have been from the third wave of behavioral therapies or third generation therapies (TTG) [98,99]. As well as acceptance-based therapies, compassion training and distress tolerance could effectively address misophonia [100,101], as well as mindfulness therapies, acceptance, and commitment therapy (ACT) [24]. 

Currently, there is only one study (2021) of treatment for misophonia that has been conducted with eye movement desensitization and reprocessing (EMDR) therapy [102]. 

Third-generation therapies aim to address the emotional responses caused by misophonic stimuli [24]. However, in order to have a truly effective treatment, larger studies ruling out comorbidity and defining diagnostic protocols are needed [58].

## 4. Discussion

This study shows an extensive bibliographic analysis of the advances of the last twenty years in the area of misophonia, which has allowed to know in depth the main problems and challenges of a little-known disorder. Aspects such as its evolution, epidemiology, etiology, diagnosis, comorbidity, evaluation instruments, and treatment have been considered. As a result, it can be stated that misophonia is a little-known and poorly studied disorder, which still has many information gaps [1]. 

### 4.1. On the Concept of Misophonia and Its Impact

The literal meaning of misophonia is “hatred of sound”, and is attributed to the intense aversive response that the person with misophonia experiences to a triggering stimulus, which is usually a sound or the observation of a movement [58]. Although many research articles used this term to describe misophonia, the members of a recent Delphi study [16] “objected to including this translation in the definition as those with misophonia neither specifically feel hate nor do they necessary feel strong emotions only related to sound (i.e., some also have similar responses to visual triggers not associated with sounds, such as leg swinging)”. 

Misophonia causes an impact and deterioration in the patient’s quality of life, both in adults and in children and adolescents, according to [84,85]. Thus, in a recent study [84], the quality of life was also impoverished in a group of children with misophonia compared to those who did not suffer from this disorder, as well as contributing to the development of other problems such as anxiety and obsessive-compulsive disorder. The most common symptoms are anger, discomfort, disgust, anxiety, and avoidance and escape behaviors [6]. Studies indicated that this is an exaggerated response to a specific stimulus, a criterion that is also shared by people suffering from misophonia, despite the fact that they tend to attribute intentionality to the people who emit the sounds [55]. One case study described a patient’s pressing need to become deaf to stop suffering from sounds [103]. Different authors highlighted the need to unify the criteria for diagnosing misophonia through validated scales and with the support of the DSM-5 [104], where the disorder is currently not covered [47,57,69]. However, despite the scarcity of studies, in recent years, there has been a significant increase in the number of studies. The reasons for their growing interest were: (1) the need to recognize that there are stimuli that may seem harmless to the general population, but that, nevertheless, in some people cause severe symptoms that considerably affect their lifestyle [2,6,20,29]; (2) the importance of correctly diagnosing the disorder without the risk of being confused with other similar conditions; [5,20,34,83]; and (3) the existing insufficiency of effective treatments that can be validated to treat the symptoms of misophonia [88].

### 4.2. The Diagnosis of Misophonia

An important area to highlight has been the interest in clarifying the diagnosis of this disorder. Due to this, some authors have focused on presenting a series of diagnostic criteria to make it valid and generalizable. Despite this, there is currently no consensus on the criteria to establish its diagnosis. Schröder et al. [20] and Dozier et al. [29], based on the body of knowledge studied to date, proposed a series of criteria for diagnosis. Building on these studies, other works incorporated variables that would gradually shape the diagnostic criteria for misophonia [2,5,28,29]. It should be considered that the model proposed by Dozier et al. [29], which was an adaptation of the one suggested by Schröder et al. [20], is undoubtedly a promising model, which if proven effective would lay the groundwork for the development of a future consensus of diagnostic criteria for misophonia among the medical-healthcare community. The summary of such criteria is summarized in the following points:

1. The presence or anticipation of a specific sensory experience such as a sound, vision or other stimulus of any sensory modality, intensity, and frequency.
2. The triggering stimulus must be a conditioned stimulus.
3. The stimulus of moderate duration (15”) elicits an immediate, reflexive, physical response.
4. Dysregulation of potentially aggressive emotions and thoughts, recognizing these as illogical and negative.
5. Avoidant and flight behaviors interfering in the person’s life.

In spite of all of them being diagnostic criteria, mostly accepted, it should be noted that in order to reach a correct diagnostic criterion, it is necessary to carry out methodological improvements such as increasing the size of the samples in the case studies, including participants from different countries and different social classes, taking into account the clinical history of the patients, and above all considering criteria that are specifically designed for this disorder [20].

### 4.3. The Differential Diagnosis and the Comorbidities

As described above, no consensus has been reached to establish diagnostic criteria for misophonia, but also no unification of criteria exists for the comorbidity in misophonia which is high and therefore a very important issue [20,29]. Thus, in recent years, it has been observed that misophonia can be confused with other disorders, both psychiatric [5,8,20,34,40,105] and auditory disorders [5,20]. For example, the symptoms of misophonia have enough similarity with features of obsessive-compulsive disorder [20,41,58,61,106]. This is noteworthy, since one of the most frequently associated disorders with misophonia is obsessive-compulsive disorder (OCD) [20,31,40,41,57,58,105,107]. Another of the most frequent comorbid disorders is anxiety [5,20,31,34,60,108] and depression [5,6,31,108]. It should be noted that different authors found that misophonia also co-occurred with other pathologies such as attention deficit disorder with and without hyperactivity (ADD/ADHD) [40,105]. Comorbidity was also observed with neurological disorders such as Tourette’s syndrome and the auditory system [41,59,66,105] and with the autism spectrum; one study, with a sample of 779 subjects, confirmed that 74% of people diagnosed with misophonia had autism spectrum conditions in 14 (3%) [105].

Regarding auditory disorders, misophonia shares qualities with auditory conditions such as tinnitus [2,40] and hyperacusis [3]. 

To have a better understanding of the causes of misophonia, it should be taken into account that there may be individual differences that determine that only a group of people develop this aversion to sounds. This specificity of stimuli and symptomatology could be due to neurobiological alterations [1,2,10,32,39,45,46,47,48,49], and/or genetic predisposition [20,23,40,41,43], which, in conjunction with learning or conditioning [3,4,24,35,52,53,56], result in the development of misophonia. From this it can certainly be concluded that in order to understand the etiology of this disorder, it is essential to approach it from a multifactorial perspective. 

In summary, the high comorbidity that misophonia has with other disorders has contributed to its underdiagnosis, as it is often confused with other psychiatric and auditory conditions [9,34]. Therefore, an important first step is to make a complete psychiatric evaluation to understand the type of reactions experienced by the patient, their intensity, the individual’s level of consciousness, the type of triggering stimuli, and the course of symptomatology [39]. It is essential to make a correct differential diagnosis, but it is also important that the clinical community and the general population are aware of the existence of this disorder. In addition, knowing the similarities and differences of misophonia with other psychiatric disorders, as well as understanding the causes of misophonia, will make it possible to more accurately delineate misophonic symptomatology and develop effective diagnostic and treatment strategies.

### 4.4. Instruments and Tools

A major advance for misophonia in recent years has been the assessment instruments and procedures, even though these remain scarce and with various methodological limitations. Undoubtedly, for most professionals, the anamnesis remains an essential resource for the assessment of misophonia [2,14,24,35,36,37,39,71]. Although the structure and content of the anamnesis are variable and depend on the needs of the professional, there are a number of data that should be collected: (a) family history [20,23,40], (b) triggering stimuli [2,27,67], and (c) behaviors or responses and coping strategies [23,35].

The creation of administered self-assessment questionnaires has been one of the great advances in recent years. While it is true that nonvalidated questionnaires and scales have been used, these have been an important resource for the study of misophonia, the most representative in chronological order being: 

(1) Misophonia Activation Scale (MAS-1) measures self-reported severity of the trait [76]. (2) Amsterdam Misophonia Scale (A-MISO-S) measures anxiety caused by misophonia and coping mechanisms [20]. (3) Misophonia Questionnaire (MQ) extensively details information about misophonia [5]. (4) Misophonia Physical Sensation Scale (MPRS) focuses on physical sensations and responses [80]. (5) Selective Sound Sensitivity Syndrome Scale (S-Five) examines emotions, behaviors, experiences, and appraisals [30]. (6) MisoQuest has been validated and published in peer-reviewed journals. It is based on the diagnostic criteria proposed by Schröder et al. in 2013. It did not include criteria such as misokinesia or exposure time, nor intensity to the misophonic trigger, among others [81]. (7) The Misophonia Response Scale (MRS) is a test of simple application. Its objective was to determine the magnitude of the misophonic response in the presence of innocuous stimuli. It showed adequate discriminant and convergent validity, with good internal consistency [82]. (8) Duke Misophonia Questionnaire (DMQ) is a broad scale with subscales. Depending on the needs, it can be applied in its entirety or only to the individual subscales [83]. (9) The first child/adolescent scale for misophonia Sussex Myofonia Scale for Adolescents SMS Adolescent [84].

An historical assessment of misophonia has been the audiological assessment, for which there is no agreement on the protocol to be applied [24]. However, although the audiological evaluation does not confirm misophonia in itself, it can be important to make the differential diagnosis of other auditory conditions with which it frequently coexists, such as hyperacusis and tinnitus, so that in these cases, the evaluation would be highly indicated [2,4,40]. The last study with this type of measurement is from 2021, and its authors suggested this online psychoacoustic test as a reliable and rapid test for the measurement of misophonia [72].

In summary, the assessment of misophonia, through self-administered inventories, needs to reinforce efforts to have valid and reliable instruments. It is also necessary to increase the sample sizes of studies based on the generalization of results, as well as to translate and adapt the scales to different populations. This does not necessarily indicate that the existing instruments are inefficient, but rather that they require greater scientific support in order to be able to trust the results they yield. However, since these questionnaires are all that are currently available, we could use them to assess the presence and severity of misophonia in patients, but with great care and attention to the interpretation and generalization of the results. Given the increase in research on misophonia, it is essential to have rigorous psychometric and multimodal tools to measure this disorder. 

Moreover, it is important to continue with research and to propose different methods of diagnosis of misophonia that allow differentiation from other conditions and, therefore, to establish valid and effective treatments to treat the symptoms of misophonia. The causes of these conditions differ, so they should be treated as distinct entities that require specific treatment [27,56]. 

### 4.5. Treatments

Regarding the work done on the treatment of misophonia, although there are currently no validated treatments, it cannot be denied that significant progress has been made in this area as well. Therapeutic interventions have been studied and proposed from audiological, pharmacological, and cognitive-behavioral techniques. 

From the audiological approach, tinnitus retraining therapy (TRT) aims to achieve habituation to misophonic sound [3,4,27,56]. Another alternative is the use of sound devices, which are the size of a hearing aid, with the aim of helping to mask the misophonic sound [69].

From pharmacological therapies, antidepressants and anxiolytics have been used to attenuate the symptomatology [24]. Other drugs such as methylphenidate [90] and propranolol, a β-blocker [33], have also been used to attenuate misophonic symptoms. 

Regarding the third block of treatments, those related to cognitive-behavioral therapies have been widely used showing that they can help to decrease misophonia symptoms and improve the patient’s quality of life [14,56,87]. The main goal of these therapies has been the development of coping skills, in general [109]. Within these therapies, educational, self-management, cognitive restructuring, and educational techniques and strategies have been used [22]. Additionally, within cognitive-behavioral therapies, we can find therapies called third generation therapies (TTG) [98,99] where work has been developed, among others, related to eye movement desensitization and reprocessing (EMDR) [101]. 

## 5. Conclusions and Future Directions

Both large sample studies and individual case studies have provided valuable information that allows for the advancement, development, and maintenance of misophonia. In the more than 20 years of studying misophonia, we have been able to accurately describe its symptomatology, approximate epidemiological data, develop explanatory hypotheses, and propose treatment options. However, it is a subject that is still in its early stages, as it is still necessary to reach a consensus on the diagnosis of the disorder, to have more assessment instruments, and even to define the nature of misophonia. Certainly, individual and collaborative efforts to summarize and integrate the knowledge on misophonia achieved since the identification of the disorder are in good pace. The growing evidence supports misophonia as an emerging field, and experts in the field encourage new research efforts to contribute to advances in understanding the nature and features of misophonia [110,111], which still needs to be recognized as a clinical entity and all efforts come short in achieving this status.

Recent advances in the treatment of misophonia allow to conclude that researchers and scientists have done a great job in proposing and studying innovative and creative treatments to treat a disorder that still does not have its clinical basis well established. Although these treatment options have not yet been validated, each has presented some improvements or advantages that should be considered in order to unify a treatment that successfully treats the symptoms of misophonia.

In the case of sounds, the most frequent trigger stimulus, we proposed our current line of therapeutic research with the development of active noise-canceling headphones. Such headphones incorporate a microphone that detects the original sound and a loudspeaker to create a phase-change sound that cancels the original sound, thus creating a soundless environment suitable for people with misophonia. This system is in an experimental phase and presents some difficulties in the kHz of certain sounds. This is certainly a palliative measure and not a treatment, but the development of hearing aids that mute annoying sounds can be of great help while research into the etiology and treatment of misophonia is progressing. 

At the same time, we consider that a multidisciplinary approach to the treatment of misophonia is desirable to reduce the misophonic symptoms: third generation therapies, virtual reality therapies, as well as the use of drugs, in situations of need, can help people with misophonia to have a better quality of life for the time being. Within the therapeutic actions, it should be considered to complete the intervention in a combined way: (1) carrying out individual activities contemplated by the specialty in question, (2) at the same time group activities are carried out where the relatives of the patients are included with the aim of supporting the person with misophonia and the family itself, and (3) educational training programs for misophonia. We consider of special interest this mixed approach to help both people suffering from misophonia and their relatives, who in parallel, also suffer from it. 

We also consider necessary the construction of a database of sound, visual, kinesthetic, and/or olfactory stimuli triggering misophonia for a faster and better identification of potential stimuli, and personalized interventions to prevent, alleviate, and relieve them from the experience. In addition, it may also be of general interest because, as Savard indicated in a recent study, the specificity of affective responses in misophonia depends on the identification of the trigger [112]. 

Despite the gaps that still exist on this topic, one thing that can be done to help people suffering from this disorder is to keep medical personnel informed of all the advances in symptomatology, diagnosis, and treatment of misophonia. In addition, patients need to be informed about the paucity of measurement tools, the lack of clarity about the etiology of the syndrome, and the lack of proven effective treatment. Ultimately, it is important to be aware of the methodological limitations of the studies in generalizing results and making assertions. Moreover, above all, it is necessary for the medical team of neurologists, audiologists, occupational therapists, neuropsychologists, clinical psychologists, and psychiatrists to maintain an open and collaborative dialogue, which will expand the knowledge and treatment of misophonia by focusing on the specific needs of each patient with misophonia.

Finally, since misophonia is considered by different authors as multisensory (sound, kinesthetic [1,5,50,105], olfactory [82], etc.), its meaning should be revised since the etymology of the word misophonia only reflects the discomfort due to sounds. We are currently building a data base of trigger sound, visual, kinesthetic, and/or olfactory stimulus that will also show the weighted incidence of the different types of triggering stimuli for an individual and allow a faster and better identification of potential stimulus, as well as the design of tailored interventions to prevent, alleviate, and relieve the individual from the misophonic experience.

## Figures and Tables

**Table 1:** Disorders most frequently involved in the differential diagnosis of misophonia.

Disorder		Similarities with Misophonia	Differences with Misophonia
Psychiatric disorder	Specific phobia	A triggering stimulus may evoke a negative response, as well as avoidance behaviors.	In specific phobia, one experiences mostly anxiety and fear, whereas with misophonia a high degree of anger and aggression is perceived [20].
	Phonophobia	Fear of a specific sound.	The main symptom is not anxiety or fear as in phonophobia, but the feeling of irritation, disgust, or anger [10,20].
	Social phobia	Habitual avoidance of social situations, due to experiencing anxiety and stress.	In social phobia, the reason is a hypersensitivity towards negative social evaluation; whereas in misophonia, the social situation is avoided to prevent an encounter with the misophonic sound [20].
	Post-traumatic stress disorder (PTSD)	Aversive reaction to a stimulus and avoidance behaviors are shared.	The person with PTSD had to experience a traumatic event. In the case of misophonia, no such association has been demonstrated [20,35].
	Obsessive-compulsive disorder (OCD)	They share an excessive preoccupation towards a specific stimulus, as well as the feeling of anxiety.	People with OCD often perform compulsive behaviors to reduce anxiety. In their case, there are no behaviors such as aggression and anger that may occur in misophonia.
	Intermittent explosive disorder	Here, the shared factor is anger.	In misophonia, the triggering stimulus of anger is always a sound, for explosive disorder it can be any stimulus. Loss of control does not usually occur in misophonia [20].
	Eating behavior disorders	The most frequent emotional trigger in misophonia, as in the TCA, is food.	For the person with misophonia, the trigger is the sound of food, for ED, it is the ingestion of food [23].
	Obsessive compulsive personality disorder (OCPD)	The shared factors are anger or aggression.	People with misophonia respond to the same auditory stimulus, people with OCPD do not relate to triggering sounds [20].
	Autism Spectrum Disorders (ASD)	Auditory hyper-reactivity is observed.	People with ASD show intolerance to unexpected and loud noises. People with misophonia can react to any type of auditory stimulus [20].
	Sensory processing disorder (SPD)	Auditory hyper-reactivity is observed.	The person with TPS reacts to unexpected and loud noises, there is also hyper-reactivity to other stimuli. The person with misophonia reacts to any auditory stimulus [20].
	Personality disorders with impulsive aggression	Difficulty in controlling anger and impulsivity occurs.	The reactions are not necessarily related to a specific sound, as is the case with misophonia [55].
Auditive disorder	Tinnitus	Can provoke negative emotions; anxiety.	It is perceived in one or both ears in the absence of acoustic source [9].
	Hyperacusis	Negative reaction to any auditory stimulus with physical characteristics (loudness and frequency) [55].	The stimulus characteristics are neutral or of very low frequency and intensity and context-independent [3,56].

**Table 2:** Self-administered scales for Misophonia.

Self-Administered Scales for Misophonia			
Name	Description	Aim	Comment
Misophonia Activation Scale (MAS-1) [76]	Developed by Fitzmaurice (Misophonia-uk-org)	To evaluate the severity of misophonia on a scale of 1 to 10 where extreme 10 indicates the most severe level of misophonic reaction.	It has been used in different studies, but has not been validated. [35,53,77]
Amsterdam Misophonia Scale (A-MISO-S) [20]	It is adapted from a scale for measuring obsessive-compulsive disorder (Yale-Brown Scale).	To measure the intensity of misophonia and its influence on social functioning, anger, and efforts to inhibit aggressive impulses.	Designed from another scale that measures another symptomatology.
Misophonia Questionnaire (MQ) [1]	It is composed of the symptom scale, the emotions and behaviors scale, and the severity scale.	To assess the sensitivity to sounds, explores the emotional and behavioral reaction associated with the stimulus.	Widely used [31,57,78,79].
Misophonia Physical Response Scale (MPRS) [80]	Developed by The Misophonia Treatment Institute	To collect the physical sensations related to misophonia.	It assesses the intensity of the physiological response.
Online questionnaire [40]	Designed to know the frequency of misophonic people in the general population.	To inquire about the family history of misophonia, the presence of symptoms, and the characteristic of the response.	
Selective Sound Sensitivity Syndrome Scale (*S*-Five) [30]	For its development, a sample was used in the United Kingdom for people over 18 years of age.	To evaluate internalizing and externalizing appraisals, as well as threat perception and avoidance behavior.	Under development and in the process of validation.
MisoQuest [81]	Validated questionnaire based on diagnostic criteria proposed in other studies.	Rule out the consideration of violent behavior in response to misophonic triggers as a symptom of misophonia.	The reliability of this questionnaire was shown to be excellent.
Misophonia Response Scale (MRS) [82]	This scale was not conducted as a specific tool to diagnose the presence of misophonia.	To know the magnitude of the misophonic response in the presence of innocuous stimuli. Auditory, olfactory, visual, and tactile stimuli are also assessed.	Adequate discriminant and convergent validity. Good internal consistency.
Duke Misophonia Questionnaire (DMQ) [83]	Scale of 86 items: subdivided into (a) general severity of symptoms and (b) coping.	Development and psychometric validation of a self-report measure of misophonia.	The subscales can be used individually.
Misophonia Emotion Responses (MER-2) [35]		At the time of the present study, these measures had not yet been subjected to psychometric evaluation.	
Misophonia Coping Responses Scale (MCRS) [35]			
Misophonia Trigger Severity Scale [35].			
Sussex Misophonia Scale for Adolescents (SMS-Adolescent) [84]		The first questionnaire of misophonia validated for children and adolescents (10 to 14 years old).	It is an adapted version of the scale for adults [85].

## Data Availability

Not applicable.
